# Potential pathogens drive ARGs enrichment during biofilms formation on environmental surfaces

**DOI:** 10.1093/ismeco/ycaf057

**Published:** 2024-04-02

**Authors:** Zihao Zheng, Zhourui Gong, Rui Zhang, Xiaoxing Lin, Wenqing Hong, Liyan Song

**Affiliations:** School of Resources and Environmental Engineering, Anhui University, Hefei 230601, China; Anhui Shengjin Lake Wetland Ecology National Long-term Scientific Research Base, Dongzhi 247230, China; School of Resources and Environmental Engineering, Anhui University, Hefei 230601, China; Anhui Shengjin Lake Wetland Ecology National Long-term Scientific Research Base, Dongzhi 247230, China; School of Resources and Environmental Engineering, Anhui University, Hefei 230601, China; Anhui Shengjin Lake Wetland Ecology National Long-term Scientific Research Base, Dongzhi 247230, China; School of Resources and Environmental Engineering, Anhui University, Hefei 230601, China; Anhui Shengjin Lake Wetland Ecology National Long-term Scientific Research Base, Dongzhi 247230, China; School of Resources and Environmental Engineering, Anhui University, Hefei 230601, China; Anhui Shengjin Lake Wetland Ecology National Long-term Scientific Research Base, Dongzhi 247230, China; School of Resources and Environmental Engineering, Anhui University, Hefei 230601, China; Anhui Shengjin Lake Wetland Ecology National Long-term Scientific Research Base, Dongzhi 247230, China

**Keywords:** microbiota, potential pathogens, ARGs, dynamics, biofilms formation, environmental surfaces

## Abstract

The enrichment of antibiotic resistance genes (ARGs) on environmental surfaces is a fundamental question in microbial ecology. Understanding the processes driving ARG variations can provide clues into their transfer mechanisms between phases and offer insights for public health management. In this study, we examined microbiota, potential pathogen, and ARG dynamics on two common environment surfaces—polyvinyl chloride (PVC) and carbon steel (CS)—under environmental stress (induced by landfill leachate flow) in a Center for Disease Control and Prevention Biofilm Reactor using metagenomics and quantitative polymerase chain reaction-Chip techniques. Contrary to the expected changes in biofilms morphology and physiochemical properties, microbiota, potential pathogens, and ARGs exhibited a divergence-convergence pattern, primarily shaped by attachment surface properties and, subsequently, biofilm maturity during biofilms formation. During this process, ARG levels in biofilms gradually increased to and exceeded the levels in the surrounding environment, but with a distinct structure (*P* < .05). Furthermore, 1.93- and 3.05-fold increases in the concentrations of mobile genetic elements intI-1 in PVC and CS biofilms, respectively, suggested their important role in the transfer and spread of ARGs within the biofilm matrix. Although potential pathogens were less abundant (3.48%–5.63%) in the biofilms microbiota, they accounted for 18.28%–45.16% of the ARG hosts and harbored multiple ARGs. Pathogens significantly impacted ARG enrichment (Procrustes analysis: *P* = .0136, M^2^ = 0.34) although microbiota development also influenced this process (*P* = .0385, M^2^ = 0.67). These results suggest that pathogens are key in shaping ARG enrichment in biofilms. Our findings provide dynamic insights into resistome enrichment on environmental surfaces.

## Graphical Abstract

**Graphical Abstract:**
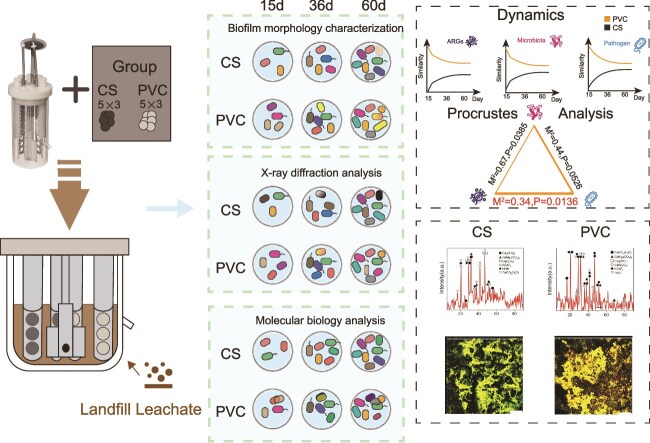

## Introduction

ARGs are a global concern, with antibiotic resistance implicated in 100 000 deaths annually and projected to cause 10 million deaths per year by 2050 [1]. ARGs are prevalent in diverse environmental settings, including natural waters, sediments, sewage, agricultural runoff [2], and biofilms [3]. Previous studies have indicated that biofilms serve as reservoirs for ARGs and antibiotic-resistant bacteria, with horizontal gene transfer (HGT) enhancing ARG content within them [4]. For instance, biofilms on marine microplastics (MPs) travel from source to sink, developing unique microbial communities with high ARG loads [5]. Similarly, biofilms at the Yangtze Estuary and its coastal zones have been identified as active sinks for antibiotics and ARGs [6].

To date, most studies on ARGs in biofilms have focused on their occurrence and prevalence on different types of biofilms [7–9]. However, the dynamics of ARGs during biofilm formation are not well characterized, limiting the understanding of the role of biofilms in ARG enrichment and dissemination. Few studies have investigated ARG dynamics in plastic-based biofilms. Xiao et al. study demonstrated that different ARGs exhibited distinct dynamic changes on various attachment materials [10]. Chen et al. conducted a biofilm incubation experiment with MPs in the Qinhuai River. Their results indicated that initially, the MP type predominantly influences the dissemination of ARGs, whereas during the later stages, the impact of water depth progressively increased [11].

ARG enrichment and dissemination are associated with the microbial community formation [12]. Therefore, elucidating ARG enrichment in biofilms must be based on an understanding of biofilm microbiota formation. Previous studies have investigated the microorganism formation and colonizer spatiotemporal development during biofilms maturation [13]. However, these pioneering works have typically employed single and/or limited species to investigate the associated mechanisms [13]. To understand the linkage between microbiota and ARGs in biofilms, the entire microbial community colonization dynamics is fundamental to understanding ARG evolution. In addition, many potential pathogens within the microbiota serve as ARG hosts [14], significantly contributing to the dissemination of ARGs through HGT. The relationship between pathogens and ARGs is crucial, as the presence of resistance genes in pathogenic bacteria can significantly complicate infection management and control. This, in turn, contributes to the persistence and dissemination of resistant infections in the environment. This dissemination process takes place in various environments, including soil, wastewater treatment systems, and natural water bodies [15–18]. Therefore, analyzing the microbiota and potential pathogen dynamics during biofilm formation is critical for understanding ARGs enrichment and risk management.

Surfaces physical, chemical, and biological properties are all critical for microbial colonization [19]. Microbial communities thrive by forming biofilms on these surfaces, enhancing their survival by increasing nutrient availability and resistance to environmental stresses. This process can negatively impact the ecosystem and public health; for instance, microbial colonization on synthetic materials used in space stations, such as rubber seals [20], viewing windows, and other hardware [21], poses serious health risks to astronauts. Similarly, the contamination of river biofilms through pharmaceutical discharges from wastewater treatment plants accumulate pharmaceuticals and foster ARGs, potentially harming human health [22].

Therefore, this study aimed to (i) characterize the dynamics of microbiota formation on two representative environmental surfaces, polyvinyl chloride (PVC) and carbon steel (CS), (ii) study the dynamics of potential pathogens formation and ARGs enrichment, and (3) link the microbiota, potential pathogens, and ARGs using metagenomics and quantitative polymerase chain reaction (qPCR)-Chip techniques. To achieve these goals, we used a 1-L CDC biofilm reactor to simulate the formation of landfill leachate biofilms on PVC and CS surfaces, representing two of the most frequently exposed environmental surfaces in public facilities. Landfill leachate biofilm provides an ideal example for this type of study. Landfill leachate is well known as an environmental stressor, with high concentrations of chemical contaminants and heavy metals [23]. A prior study indicated that landfill leachate acts as a significant reservoir and refuge for ARGs and potential pathogens, highlighting its role in ARG dissemination and, as well as disease transmission [24].

## Materials and methods

### Leachate sampling and experiment design

Raw leachate was obtained from the landfill leachate collection system of a landfill in Anhui Province, China. Samples were collected in a 100-L sterilized PVC bucket and stored on ice. They were then sent to the laboratory within 24 hours and stored at 4°C for further analysis.

To investigate microbiota colonization and ARGs enrichment during biofilms formation, a 1-L CDC biofilm reactor (Biosurface Technologies, Inc., Bozeman, MT) with a coupon holder and 10-mm-diameter coupons of PVC and CS was used to generate leachate biofilms. The CDC biofilm reactor operation followed the ASTM 2562-07 standard (Standard Test Method for Quantification of *Pseudomonas aeruginosa* Grown with High Shear and Continuous Flow Using a CDC Biofilm Reactor). Briefly, the reactor included a 1-L vessel (335-ml capacity up to the outlet), a magnetically driven baffle stirrer, a MASTERFLEX L/S peristaltic pump, and eight UHMW polyethylene sampling rods with removable PVC and CS coupons (15 pieces each).

Based on preliminary experiments, a 60-day experiment was conducted, which involved passing leachate through the system twice daily (8:00–9:00 a.m. and 8:00–9:00 p.m.) at a flow rate of 5.6 ml/min, with the magnetic stirrer operating at 60 rpm. Five replicate samples of PVC and CS coupons were collected on Days 15, 36, and 60, and labeled as P-15, P-36, P-60, C-15, C-36, and C-60, respectively. One sample from each group was used for biofilm morphology characterization using confocal laser scanning microscopy (CLSM; Leica, Germany). One sample was used for X-ray diffraction (XRD) analysis to determine its mineral crystal phase composition. Triplicate samples were used for molecular biology analysis.

### Leachate and biofilms genomic deoxyribonucleic acid extraction

Following the manufacturer’s instructions, DNA was extracted from leachate and leachate biofilms using the PowerSoil DNA Isolation Kit (MoBio, USA). DNA concentration was determined using a Nanodrop ND-1000 spectrophotometer (Thermo Fisher Scientific, USA), and 1% agarose gel electrophoresis was performed to assess the mass and integrity of the isolated DNA. The isolated DNA was stored at −80°C for further sequencing analysis.

### Metagenomic sequencing and assembly

A total of 21 samples were sequenced on an Illumina HiSeq 4000 platform (Illumina, Inc., San Diego, CA, USA) at Majorbio Bio-Pharm Technology Co., Ltd. (Shanghai, China) using HiSeq 3000/4000 PE Cluster Kits and HiSeq 3000/4000 SBS Kits. Each sample generated 7 GB of raw data (Table S1). After removing low-quality sequences and trimming adapters with Fastp (v0.20.0, https://github.com/OpenGene/fastp), the clean sequences were assembled into contigs using Megahit (v1.1.2, https://github.com/voutcn/megahit). Prodigal (v2.6.3, https://github.com/hyattpd/prodigal) was then used to predict open reading frames (ORFs) from the contigs. For species identification, the predicted ORFs were clustered using CD-HIT (http://www.bioinformatics.org/cd-hit/) with 95% identity and 90% coverage, selecting the longest gene in each cluster to construct a non-redundant gene set. High-quality reads were then aligned to the non-redundant gene set using SOAPaligner (http://soap.genomics.org.cn/) to calculate gene abundance, and taxonomy annotation was performed by aligning the non-redundant gene set with the NR database using DIAMOND (e-value = 1e^−5^). For ARG identification, the DeepARG pipeline (v1.0.2, https://bench.cs.vt.edu/deeparg) with default parameters (minimum identity of 20%, E-value <1e^−10^, maximum 10 000 alignments) [25] was used to predict ARGs from the ORFs. Contigs containing ARGs were further classified taxonomically using the Contig Annotation Tool (CAT, v5.2.3, https://github.com/dutilh/CAT) [26] to identify potential ARG hosts.

### Pathogen annotation and human pathogenic bacteria list construction

To identify potential pathogens, taxonomy annotation was first performed on the 21 samples, with names updated based on the latest information from the NCBI taxonomy database (https://www.ncbi.nlm.nih.gov/taxonomy). By reviewing literature and databases [27–31], we made a list of human pathogenic bacteria (HPB) with the species name. Species with standardized nomenclature, corrected errors, and updated names from the NCBI taxonomy database were retained, resulting in a final HPB list of 848 species. The list has been uploaded to https://github.com/hongwenqing/HPB-identification-principle and also is available in the Supplementary Information Dataset 1.

### Metagenomic binning

Metagenomic binning processing begins by filtering the metagenomic contigs by length. Following this initial selection, binning assembly is performed using MetaBAT [32] (version 2.12.1), CONCOCT [33] (version 0.5.0), and Maxbin2 [34] (version 2.2.5). The bins produced by these software tools are then integrated using DAS Tool [35] (version 1.1.0), which refines and merges bins across different platforms to generate a consolidated set. These refined bins are further purified using RefineM (Version 0.0.24). Quality assessment of the bins is conducted using CheckM [36] (Version 1.0.12), where only bins with completeness ≥50% and contamination <10% are retained. Subsequent deduplication and clustering are carried out using dRep (Version 3.4.2). Species annotation for the bins is performed using GTDB-TK (Version 2.3.0). Finally, genes assembled from the genomes are aligned against the CARD [37] database using Diamond software, with a set expectation value (E-value) of 1e^−5^ for the alignments. The reconstructed MAGs information is provided in Table S2.

### Quantification of antibiotic resistance genes and mobile genetic elements by quantitative polymerase chain reaction

To quantify abundant biofilms ARGs (sul1, sul2, and ermF) and potential pathogen (*Campylobacter jejuni*), high-throughput quantitative PCR (HT-qPCR) was performed using the WaferGen SmartChip Real-Time system in Hefei Yuanzai Biotechnology Co., LTD, China. In addition, The mobile genetic element (MGE) intI-1, a crucial element for ARG distribution within biofilms was also quantified. The associated primers were provided in Table S3. The system is capable of running 5184 nanoliter-scale reactions in parallel [38]. The system operation and PCR condition can be seen at Supplementary material and method 1. The relative copy number of a gene was determined using the following formula: gene relative copy number = 10^(31-Ct)/(10/3)^, where Ct represents the threshold period for each gene. HT-qPCR data were further analyzed to ensure consistency and replicate reliability, following the procedures outlined in previous study [39].

### Determination of physicochemical properties in leachate and leachate biofilm

Raw leachate physiochemical parameters [chemical oxygen demand (COD), biological oxygen demand (BOD_5_), total nitrogen (TN), total phosphorus (TP), ammonium-N] were analyzed using standard methods for water and wastewater examination [40]. Extracellular polymeric substances (EPS) were quantified using spectrophotometric methods. Nucleic acids were measured at absorbances of 260 and 280 nm [41]. Polysaccharides were quantified using the phenol-sulfuric acid method, with absorbance measured at 490 nm [42]. Protein content was determined using the Coomassie Brilliant Blue method, with absorbance read at 595 nm [43]. To ensure accuracy, each measurement was calibrated against standard curves of known concentrations. Dissolved oxygen (DO) concentration, redox potential, conductivity, and pH were determined using a portable multiparameter water quality analyzer (Hach, Loveland, CO, USA).

Leachate-formed biofilms morphology were observed using CLSM. The biofilm samples were rinsed and fixed on glass slides using PBS buffer. The fixed biofilms were stained with LIVE/DEAD® BacLight™ (Thermo Fisher Scientific, USA) stain for 50 minutes in the dark. Subsequently, the biofilms were examined using laser confocal Z-stack mode, and the 3D biofilms structure was reconstructed using Imaris software (https://imaris.oxinst.com). The chemical properties of the biofilms were determined via grazing incidence XRD (GIXRD; Malvern Panalytical, Netherlands). The data obtained were analyzed using the Jade software (https://www.materialsdata.com; Version 6.5).

### Data analysis

Microbial community and ARGs compositions obtained from metagenomic sequencing were presented in terms of reads per kilobase of transcript per million mapped reads (RPKM). The ratio of potential pathogens to detected microbes and ARGs abundance (for qPCR) were expressed as mean ± standard deviation (SD). To compare differences among groups, one-way analysis of variance (ANOVA) was conducted, followed by Tukey’s honestly significant difference (HSD) test for multiple comparisons, with a significance level of 0.05 and group differences indicated using Tukey HSD markers (e.g. a, b, c). PERMANOVA (Adonis test with 999 permutations, using the vegan package in R) was used to determine the differences in microbial communities and ARGs, as well as the contributions of time and attachment materials to these variations. Spearman rank-order correlations among species were calculated using the psych package, and significant relationships in the co-occurrence network were visualized using Gephi. Procrustes analysis was employed to assess the relationship between microbial communities and ARGs. Bubble charts were created using ggplot2, and UpSet plots were used to present shared ARGs. The significance level was set to *P* < .05.

## Results

### Morphological and physiochemical properties of biofilms

Landfill leachate physiochemical parameters were shown in Table S4. Leachate had high concentrations of TN (2413.1 mg/L), TP (6.0 mg/L), ammonium nitrogen (2366.0 mg/L), COD (1972.6 mg/L), suspended solids (156.5 mg/L), conductivity (27.2 mS/cm), and EPS (75.8 mg/L; Table S4). It also exhibited a low oxidation–reduction potential (−193.7 mV). The DO concentration was 0.2 mg/L, and the pH was 8.0. BOD_5_ was 55.1 mg/L, indicating low biodegradability of the leachate.

**Figure 1:**
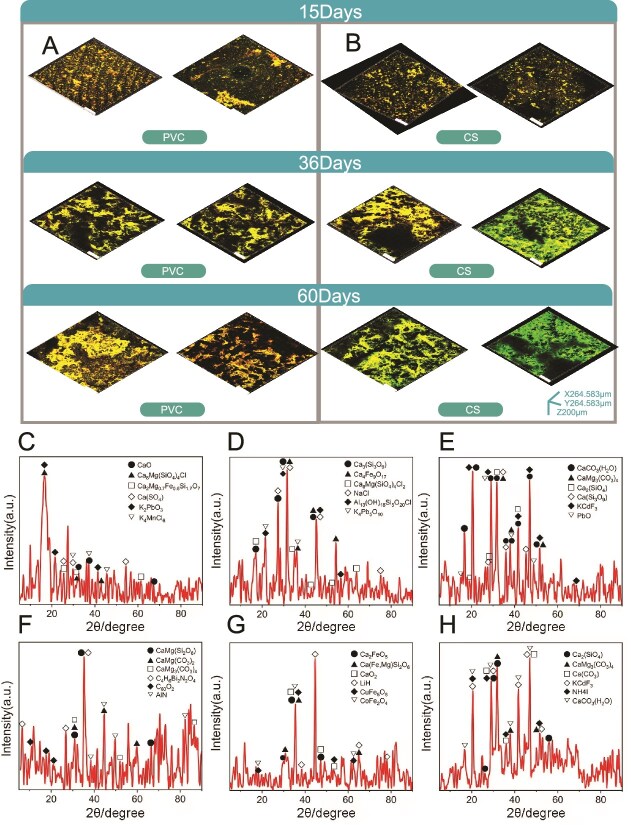
Biofilms morphology and chemical composition dynamics during the formation. (A) Structural changes in biofilms over different developmental stages. (B) XRD patterns of leachate biofilms. Patterns are shown for (C) P-15, (D) P-36, (E) P-60, (F) C-15, (G) C-36, and (H) C-60, highlighting the structural differences at different stages.

Biofilms formation varied across different stages on PVC and CS surfaces (Figs 1A and B), as evidenced by increasing biofilms thickness and EPS values. Biofilms EPS concentrations on PVC and CS were 64.2 mg/L for P-15, 70.4 mg/L for P-36, 70.5 mg/L for P-60, and 76.9 mg/L for C-15, 76.9 mg/L for C-36, and 93.6 mg/L for C-60, respectively (Fig. S1), demonstrating the dynamics of biofilms formation on these surfaces. PVC had lower EPS concentrations than CS, indicating that surface properties are critical for biofilms adhesion and development. PVC has higher corrosion resistance in leachate environments compared to CS, resulting in smoother surfaces that are less conducive to biofilm adhesion. Rust spots were observed on the CS surface.

XRD analysis revealed the chemical composition variation in biofilms during their formation on PVC and CS surfaces (Supplementary Note 1). The primary components were O, Ca, Mg, and Si, with trace amounts of K, Pb, Na, Al, Bi, Li, and Cu (Figs 1C–H). The distinct chemical composition of PVC and CS biofilms suggested that surface properties significantly influence biofilms chemical composition and mineralization over time (Supplementary Note 1).

### Dynamics of biofilms microbiota during biofilms formation

Microbiota development during biofilms formation on PVC and CS surfaces was analyzed and compared (Supplementary Note 2). At the phylum level, the bacterial community structures on both surfaces were similar. Proteobacteria (67.60%–85.20%), Bacteroidetes (6.08%–15.18%), and Actinobacteria (2.50%–6.32%) were the dominant phyla across all samples, constituting >80% of the total (Fig. S2).

**Figure 2:**
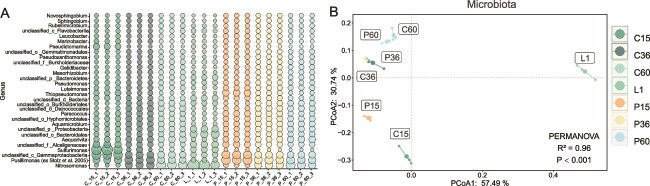
Dynamics of biofilms microbial community formation. (A) Microbial community composition at the genus level, depicted as a bubble plot of the top 30 genera in different samples on 15, 36, and 60 days of formation. (B) PCoA depicted the dynamics of microbiota. PERMANOVA results indicate significant differences among the groups (significant level: *P* < .05).

At the genus level, *Nitrosomonas* (0.38%–11.48%), *Pusillimonas* (5.35%–11.07%), and *Sulfurimonas* (0.05%–15.03%) were the most abundant groups (Fig. 2A), but exhibited different patterns on PVC and CS biofilms. *Nitrosomonas* was more abundant in leachate than in biofilms, likely owing to the high TN and ammonia-N concentrations and low DO (0.2 mg/L) favoring ammonia oxidation [44]. In early stages, *Nitrosomonas* had a lower relative abundance on CS and PVC biofilms; however, its abundance increased with biofilms maturation, suggesting an increasing role of ammonia oxidation within the biofilm. *Pusillimonas*, previously found in landfills and soil [45], was more abundant in biofilms at all stages and on both surfaces than in leachate, suggesting that the solid phase (PVC and CS surfaces) favors *Pusillimonas* colonization [46]. *Sulfurimonas* thrived in the reduced environment indicated by the leachate’s redox potential (−193.7 mV), explaining its high abundance in C-15 biofilms. Its abundance decreased over time, likely owing to sulfide depletion [47].

Principal coordinate analysis (PCoA) clearly separated leachate and biofilms microbiota at different stages into distinct groups, indicating dynamic shifts in the biofilm microbiota during maturation (Fig. 2B). PC1 and PC2 explained 89.2% of the variation. A PERMANOVA test confirmed significant contributions of surface properties and biofilm maturation to biofilm community differentiation, explaining 96% of the variance (*P* < .05). In the early stage, the difference in microbiota between P-15 and C-15 was large, suggesting that surface properties influence microbial adhesion. Over time, the microbiota of P-36 and C-36, and P-60 and C-60 became more similar, indicating that biofilm maturation plays an important role in shaping the biofilm microbiota. The microbiota of all biofilms differed from the leachate, because of the solid–liquid phase-selection effects on microbial mobility, attachment patterns, and nutrient diffusion [48].

### Co-occurrence network analysis of biofilms microbiota

**Figure 3:**
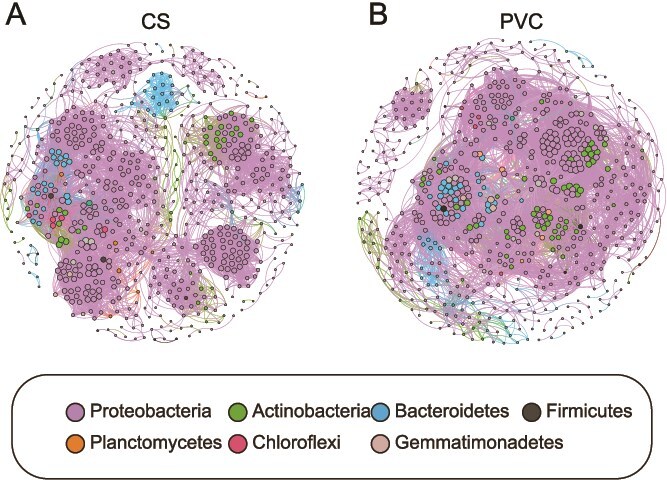
Co-occurrence network analysis of biofilms microbial communities. (A) Co-occurrence network of microbial communities in CS biofilm. (B) Co-occurrence network of microbial communities in PVC biofilm. The size of each node represents the relative abundance of the corresponding microbial taxa, and edges indicate significant correlations between taxa.

Microbial co-occurrence networks are essential for studying microbial symbiotic patterns and identifying keystone species [49]. The co-occurrence networks of the CS biofilm (+6762; −1327) and PVC biofilm (+7966; −3996) showed considerably more positive edges than negative ones (Fig. 3), suggesting synergistic interactions among the microbiota in both [50]. Positive edges can usually be attributed to two factors: niche overlap (between phylogenetically related taxa) [51] and mutualistic/facilitative interactions (between distinct bacterial taxa) [52]. Additionally, many species from different phyla appeared to assemble together in the network, supporting reciprocal/facilitative interactions (Supplementary Note 3). The graph (Fig. 3) shows that the PVC biofilm had a higher average degree (35.0) than the CS biofilm (23.4), indicating stronger microbiota interaction strength in the PVC biofilm. The modularity index is used to determine whether a network has a modular structure [53, 54]. The modularity index was 0.59 for PVC and 0.75 for CS. The average path length (3.91) and network diameter (16) for the PVC biofilm were lower than those for the CS biofilm (6.98 and 25, respectively). The average path length, a measure of network efficiency [55], and the network diameter, the longest distance between nodes, both suggest a more efficient and tightly connected microbial community on the PVC biofilm.

### Dynamics of potential pathogens during biofilms formation

A total of 478 potential pathogens were identified across all samples. Leachate samples had 368–370 pathogens, whereas biofilms had 345–381 pathogens. Most potential pathogens in biofilms showed an increasing trend over time. In the early stages of biofilms formation, the abundance of potential pathogens was relatively low (C15: 3.64%, P15: 4.11%). As the biofilms matured, potential pathogen abundance gradually increased to levels similar to those found in leachate (C60: 5.75%, P60: 5.31%, L1: 5.62%; Fig. S3), consistent with previous findings that potential pathogens abundance on MP biofilms is comparable to that in the surrounding water [10].

**Figure 4:**
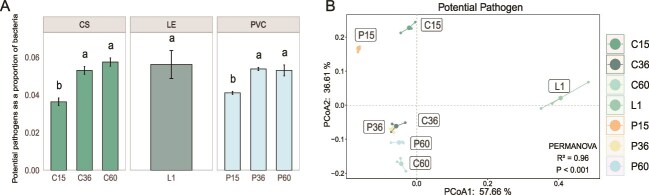
Dynamics of potential pathogens in biofilms formation. (A) Abundance of potential pathogens are taken as a proportion of total bacteria in biofilms at different stages, represented as a bar plot. (B) PCoA depicted the dynamics of potential pathogens. PERMANOVA results indicate significant differences among the groups (significant level: *P* < .05).

Dominant potential pathogens in the biofilms included *Burkholderiales bacterium* (0.45%–2.71%), *Alcaligenes faecalis* (0.45%–0.95%), and *P. aeruginosa* (0.45%–0.72%; Fig. 4A), and their colonization showed varying trends. *Burkholderiales bacterium* on CS surfaces increased significantly from 0.44% on Day 15 to 2.63% on Day 60, and on PVC surfaces from 0.53% to 2.19%. *A. faecalis*, initially more abundant on PVC (0.91%), decreased to 0.60% over 60 days, whereas on CS surfaces it decreased from 0.51% to 0.44%. *P. aeruginosa* showed little variation, with 60-day abundances of 0.68% on CS and 0.64% on PVC.

Overall, potential pathogens formation in biofilms showed a similar pattern to the biofilm microbiota. Initially, potential pathogens differed in structure and abundance in PVC and CS biofilms owing to surface selection effects. As the biofilms matured, both the composition and abundance of potential pathogens within them converged (Fig. 4B), demonstrating that biofilm maturation is crucial for shaping the potential pathogen composition.

### Dynamics of antibiotic resistance genes on biofilms formation

**Figure 5:**
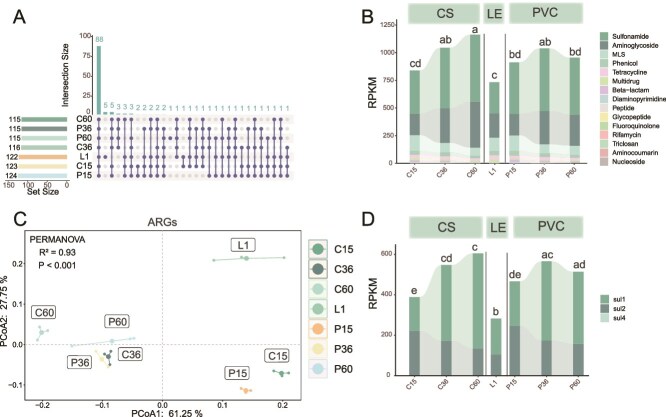
Dynamics of ARGs in biofilms formation. (A) UpSet plot revealing ARGs shared between samples, illustrating the intersections of ARGs across different stage. (B) Composition of ARGs types in PVC and CS biofilms. Different colors represent different ARG types. One-way ANOVA and Tukey’s HSD test were performed to compare the differences in ARGs abundance across different stages, with a significance level of *P* < .05. (C) PCoA depicted the dynamics of ARGs. PERMANOVA results indicate significant differences among the groups (significant level: *P* < .05). (d) Composition of sulfonamide ARGs in PVC and CS biofilms, with different colors representing different sulfonamide ARGs.

In total, we detected 15 types of ARGs encompassing 138 subtypes across all samples. The leachate samples contained 104–114 ARG subtypes, whereas the biofilms samples contained 95–113 ARG subtypes. Most ARGs (88) were shared between biofilms and leachate, with five unique to leachate [EreB, aad(6), SAT-4, cfrC, and tet (W/N/W)] and five unique to biofilms [Erm(36), mtrA, cmlA5, dfrA5, and dfrA15; Fig. 5A].

The relative abundance of ARGs in biofilms varied significantly on PVC and CS surfaces during biofilm formation (*P* < .05; Fig. 5B). Over time, ARG abundance increased steadily on CS surfaces, whereas on PVC surfaces, it reached the detected highest value at Day 36 and then slightly decreased at Day 60. During the biofilm formation process, the most abundant ARG types were those conferring resistance to sulfonamides (46.53%–55.06%), aminoglycosides (20.46%–36.37%), macrolide-lincosamide-streptogramin B (MLS) antibiotics (6.64%–16.45%), phenicols (1.90%–5.15%), tetracyclines (1.58%–3.70%), and multiple drugs (0.97%–2.51%; Fig. 5B).

PCoA revealed distinct clusters of biofilm samples from the leachate (Fig. 5C). In the early stage (Day 15), ARGs showed different compositions on PVC and CS surfaces. As biofilms matured, the ARG composition in the PVC biofilm became more similar to that in the CS biofilm. By contrast, the ARGs composition in the CS biofilm continued to vary. A PERMANOVA test confirmed that both surface properties and biofilm maturation significantly contributed to the differentiation in ARG composition, explaining 93% of the variance (*P* < .05; Fig. 5C).

Among ARGs, sulfonamides were the most prevalent group across in all biofilms (Fig. 5D), with three subtypes: sul1, sul2, and sul4. Among these, sul1 and sul2 were consistently detected across all samples, exhibiting high abundances (sul1: 19.00%–41.08%, sul2: 10.69%–27.93%). The extensive prevalence of sulfonamide antibiotics in landfills is due to the proliferation of sulfonamides ARGs [56].

Aminoglycoside ARGs were found in high relative abundances in biofilms. They are often used as potent antibiotics for severe infections caused by aerobic Gram-negative bacteria [57]. Similarly, MLS ARGs were prevalent in biofilms (Fig. S4A). They act by inhibiting protein synthesis through binding to the bacterial 50S ribosomal subunit [58]. And the rapid increase of ANT (3″)-IIa in biofilms (Fig. S4B) further supports the idea that biofilms selectively enrich certain ARGs from the leachate, suggesting a bidirectional selection process during biofilm formation and maturation. This selective enrichment emphasizes the adaptive capacity of biofilms to dynamically modulate ARG composition in response to environmental stress and community interactions [59, 60].

### Dynamics of antibiotic resistance genes, mobile genetic element, and potential pathogens during biofilms formation identified by quantitative polymerase chain

**Figure 6:**
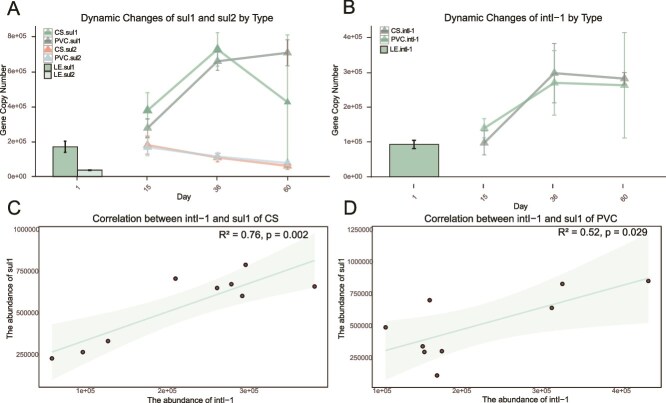
Dynamics of ARGs, MGEs, and potential pathogens during biofilms formation. (A) Dynamics of sul1 and sul2 (mean ± SD; *n* = 3) over time in PVC and CS biofilms, shown as gene copy number, the data point at Day 1 represents the gene copy numbers in leachate. (B) Dynamics of the intI-1 integrase gene across PVC and CS biofilms over time (mean ± SD; *n* = 3), the data point at Day 1 represents the gene copy numbers in leachate. (C) Linear fitting of sul1 and intI-1 in CS biofilms. (D) Linear fitting of sul1 and intI-1 in PVC biofilms.

To further characterize the dynamics of ARGs and potential pathogens during biofilm development, we examined two types of sulfonamide resistance genes (sul1 and sul2), one type of MLS resistance gene (ermF), one type of integron integrase gene (intI-1), and one type of pathogenic bacterium (*C. jejuni*, identified by the marker gene cadF) using qPCR. Sulfonamide and MLS ARGs were chosen as markers due to their high abundance in the formed biofilms in this study and in landfill and landfill leachate [56, 61, 62]. The results indicated significantly higher abundances of sul1 and sul2 compared to those of other qPCR-verified ARGs (Fig. 6A). Sul1 abundance increased 2.55-fold on the CS surface, rising from 276 780.6 ± 52 551.1 gene copies in C-15 to 705 611.7 ± 74 554.6 in C-60. On the PVC surface, sul1 abundance was the highest during biofilm formation, with 725 203.6 ± 95 524.1 gene copies on Day 36, before decreasing to 424 323.2 ± 383 255.3 gene copies on Day 60. The abundance of sul2 gradually decreased on both surfaces but remained higher than in the leachate, consistent with the metagenomic results.

The MGE intI-1, a crucial element for ARG distribution within biofilms [63], is known to play a key role in HGT and may facilitate the spread of sul1 [64]. Previous studies have used intI-1 abundance as a marker for evaluating the potential of HGT [65], as many integrons are homologous to intI-1 [66]. Therefore, intI-1 was chosen as one of the targets. It exhibited high abundance at all stages, surpassing leachate concentrations by Day 15 and increasing sharply on Day 36, especially on the CS surface, where it rose from 97219.6 ± 34316.4 to 296693.6 ± 85027.8 gene copies (3.05-fold increase). Additionally, it showed a 1.93-fold increase on the PVC surface (Fig. 6B). This high abundance suggests a greater propagation of various ARGs in high-frequency HGT scenarios. Correlation analysis between intI-1 and sul1 revealed a significant correlation (*P* < .05) on both surfaces, highlighting their symbiotic patterns (Figs 6C and D). The abundance of ermF in the PVC biofilm increased from 22 269.8 ± 1575.7 gene copies in P15 on Day 15 to 34 451.2 ± 4338.1 gene copies by P60 on Day 60, whereas its abundance in the CS biofilm remained stable and close to leachate levels (Fig. S5A), consistent with the metagenomic findings.

*C. jejuni* (Fig. S5B) [67, 68] is a human pathogenic bacterium and a leading cause of bacterial gastroenteritis [69]. We used cadF, a virulence marker for *C. jejuni*, to quantify this species. Although its abundance was low in both biofilms and leachate, the trend differed significantly between biofilms, possibly owing to their surfaces effect.

### Antibiotic resistance genes and their pathogenic hosts

**Figure 7:**
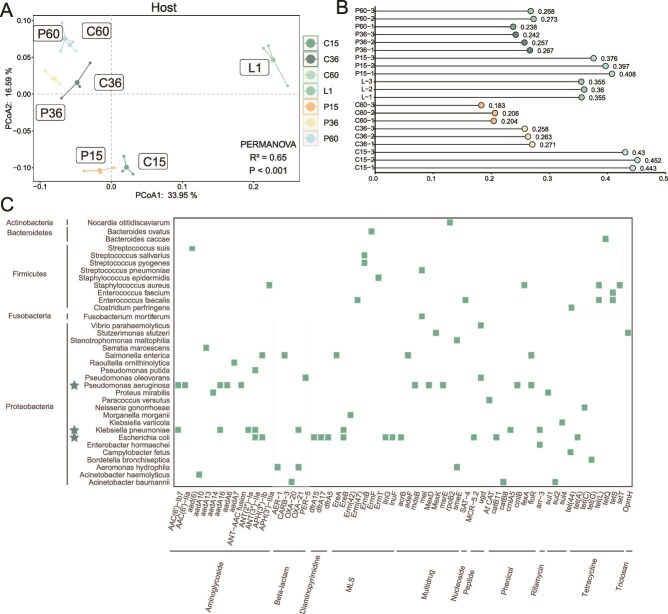
Pathogenic hosts of ARGs. (A) PCoA depicted the dynamics of host species. PERMANOVA results indicate significant differences among the groups (significant level: *P* < .05). (b) The proportion of pathogenic bacteria in hosts of various ARGs across each sample. Bar plot highlights the relative abundance of pathogenic bacteria. (c) The heatmap shows the relationship between various ARGs and their corresponding pathogenic hosts. Each cell represents the presence (deep blue) or absence (white) of a specific ARG subtype within a host species. The X-axis lists ARG subtypes, while the Y-axis lists host species, grouped by their phylum. Hosts marked with a star indicate those that harbor a higher diversity of ARGs.

Biofilms enhance the functions of microbial communities, including increased antibiotic resistance [70]. Through classification annotation of ARGs bacterial hosts, we identified 145 antibiotic-resistant species across 13 phyla. As biofilms matured, the number of host species slightly decreased (from 110 to 104 on CS and 111 to 102 on PVC from Day 15 to 60) and their dynamics can be seen at Supplementary Note 4. PCoA indicated that ARGs hosts became more similar over time (Fig. 7A), suggesting that biofilms on different materials mature to have similar ARG hosts.

We also discovered 35 pathogenic bacteria serving as ARG hosts, with 22 species harboring at least two ARGs. Although the abundance of pathogens in the biofilms was low, they contributed significantly to the ARG hosts, particularly during the early stages of biofilms formation (C-15: 0.442 ± 0.006, P-15: 0.394 ± 0.009; Figs 7B and7C). This contribution gradually decreased as the biofilms matured, although the overall content of ARGs and pathogens increased. Among the pathogen hosts, *P. aeruginosa* stood out, containing 10 ARGs, including mdsB. This pathogen exhibits strong resistance to conventional antibiotics, particularly in immunocompromised individuals such as cancer patients, surgical patients, severe burn victims, or HIV carriers [71].

### Relationships between antibiotic resistance genes and microbiota and potential pathogens

**Figure 8:**
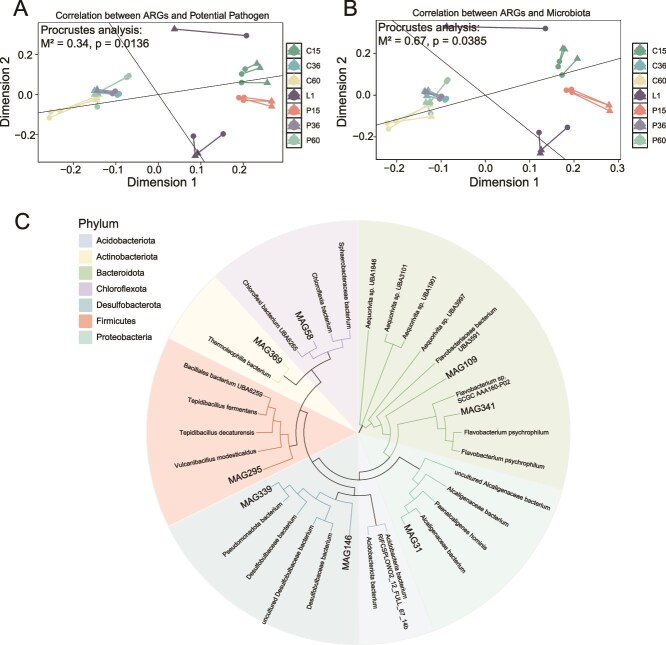
Relation between ARGs and microbiota. (A) Procrustes analysis of the relationship between ARGs and pathogens. Different shapes and colors represent different sample types, as indicated in the legend. (B) Procrustes analysis of the relationship between ARGs and microbiota. (C) MAGs phylogenetic tree. The phylogenetic tree constructed using PhyloPhlAn illustrates the taxonomic placement of MAGs at the phylum level.

Procrustes analysis revealed a significant correlation between the composition of ARGs and pathogens (*P* = .0136, M^2^ = 0.34; Fig. 8A), indicating that ARGs are closely associated with pathogens presence. Similarly, a significant correlation was observed between ARGs and the microbial community (*P* = .0385, M^2^ = 0.67; Fig. 8B), although the higher M^2^ value suggests a slightly weaker association compared to pathogens. Lastly, the correlation between microbial communities and pathogens was marginally significant (*P* = .0526, M^2^ = 0.44; Fig. S6A). These results suggest that ARGs in biofilms are more strongly influenced by pathogens than by the entire microbial community, highlighting the critical role of pathogens in the dissemination of ARGs within them.

To further investigate the potential host characteristics of ARGs, we reconstructed metagenome-assembled genomes (MAGs) from metagenomic data. We collected eight high-quality bacterial MAGs and constructed a phylogenetic tree (Fig. 8C). Analysis of the ARG composition and species classification in these MAGs revealed diverse ARGs conferring resistance to antibiotics such as tetracyclines, fluoroquinolones, macrolides, sulfonamides, peptides, and aminoglycosides (Fig. S6B). These species host ARGs information can be seen at Supplementary Note 5.

## Discussion

### Various conditions co-shaped biofilms microbiota dynamics

Environmental conditions [72], surface properties [11], and biofilm maturity [11] play crucial roles in shaping biofilm microbial community colonization, and their effects vary during the biofilm formation. The microbial community composition in biofilms is primarily derived from microbial communities in the surrounding leachate [73], and leachate physiochemical properties shape the initial microbiota. For instance, pH fluctuations can significantly influence the microbial community composition within the biofilm by promoting acid-tolerant bacteria and inhibiting others [74]. Similarly, temperature variations can encourage the growth of thermophilic organisms such as cyanobacteria while reducing populations of temperature-sensitive species, thereby reshaping the microbial community [75]. In this study, leachate features high abundances of *Nitrosomonas* and *Sulfurimonas*, which are related to high ammonia concentration [44] and redox [47], respectively. However, the distinct differences between the biofilm and leachate microbiota identified via PCoA indicate strong solid–liquid phase-selection effects on microbial mobility, attachment patterns, and nutrient diffusion [48]. Furthermore, PCoA analysis and PERMANOVA tests showed significant microbiota dynamics during the early biofilm formation stage (15 days) on PVC and CS surfaces, indicating that surface properties are imperative for shaping biofilm microbiota. As the biofilms matured, the microbiota on their surfaces became increasingly similar, suggesting that biofilm maturation becomes a dominant factor influencing the biofilm microbiota. Specifically, the biofilm microbiota formation provides example for microbial community assembly explanation and predication [76].

### Pathogens colonization showed similar pattern to microbiota

The potential pathogens colonization pattern was similar to that of the overall microbiota. Despite initial differences in composition and abundance on the PVC and CS biofilms, the potential pathogen composition converged during their colonization, indicating the combined effects of environmental conditions, surface properties, and biofilm maturation. Notably, the dominant potential pathogens-*Burkholderiales bacterium*, *A. faecalis*, and *P. aeruginosa*-showed different abundance variations during biofilm development. *Burkholderiales bacterium* abundance consistently increased, whereas that of *A. faecalis* decreased, suggesting competition between these taxa.

### Antibiotic resistance genes enrichment is similar to microbiota and pathogens colonization

Biofilms exhibit a significant capacity to enrich and concentrate ARGs [11]. Previous studies have shown that ARGs tend to be selectively enriched within biofilms [11, 77–79]. Furthermore, high-intensity interactions within the microbiota may increase the HGT frequency and enhance ARG reproduction [80, 81]. ARGs abundances in PVC and CS biofilms were higher than in the leachate, underscoring the role of biofilms in ARG accumulation. PCoA analysis showed significant differences in the ARG composition of early-stage biofilm samples based on PVC and CS surface properties, but this difference lessened over time for PVC biofilms.

Both metagenomic and qPCR analyses revealed that sul1, sul2, and intI-1 are key components in ARG distribution and enrichment. sul1 and sul2 are representative ARGs in landfill leachate because sulfonamides are among the dominant antimicrobials in landfills [82]. The integron integrase gene intI-1 significantly contributes to the distribution and expression of ARGs within biofilms [11], and in this study, intI-1 abundance increased dramatically (2.89-fold in C-60 compared to C-15 and 1.88-fold in P-60 compared to P-15) during biofilm formation. Additionally, intI-1 was strongly correlated with sul1, emphasizing the importance of HGT in ARG propagation within biofilms [63].

### Pathogens significantly impacted the antibiotic resistance genes enrichment

The presence and distribution of ARGs in biofilms are closely linked to the pathogen composition on different biofilm surfaces. Procrustes analysis underscores this relationship, with significant correlations between ARGs and potential pathogens, highlighting the crucial role of potential pathogen in shaping the ARG distribution. Pathogens significantly contribute to the spread of ARGs through HGT, further emphasizing their critical role in ARG dissemination across different ecosystems [15–18]. We identified 35 pathogenic species harboring ARGs, with *P. aeruginosa* being particularly notable owing to its high resistance and carriage of multiple ARGs. Although the early-stage contribution of pathogens to ARGs hosts decreases as biofilms mature, the overall ARGs and pathogens content increases, highlighting the potential health risks posed by pathogenic bacteria in biofilm communities.

## Conclusion

This study examined microbiota, potential pathogens and ARGs dynamics during biofilms formation on two kinds of surfaces under environmental stressor. Starting from the same initial leachate community, the microbiota, potential pathogens and ARGs exhibited a divergence-convergence pattern shaped first by attachment surface properties and then by biofilm maturation. Dramatic increase in the abundance of the MGE intI-1 in both biofilms suggests its important role in the transfer, and spread of ARGs within the biofilm matrix. Importantly, we found that although potential pathogens were less abundant (3.48%–5.63%) in the biofilm microbiota, they accounted for 18.28%–45.16% of the ARGs hosts and harbored multiple ARGs. These findings highlight the need for further analysis of microbiota colonization and ARGs prevalence on various environmental surfaces under different conditions.

## Supplementary Material

Supplementary_Information_ycaf057

Dataset_1_ycaf057

## Data Availability

High-throughput sequencing data are available in the NCBI under accession number PRJNA1108013. All other datasets are available upon request.
