# Supplementary material for: Potential pathogens drive ARGs enrichment during biofilms formation on environmental surfaces
Source: ISME Commun. 2024 Apr 2;5(1):ycaf057. doi: 10.1093/ismeco/ycaf057 (PMC12016037; doi:10.1093/ismeco/ycaf057)
Supplement: Supplementary_Information_ycaf057 [file supplementary_information_ycaf057.pdf]

Supplementary Information

Potential pathogens drive ARGs enrichment during biofilms formation on environmental surfaces

Zihao Zheng[1,2], Zhourui Gong[1,2], Rui Zhang[1,2], Xiaoxing Lin[1,2], Wenqing Hong[1,2], Liyan Song[1,2]*

[1]School of resources and environmental engineering, Anhui University, Hefei 230,601, China

[2]Anhui Shengjin Lake Wetland Ecology National Long-term Scientific Research Base, Dongzhi, 247,230, China

*Correspondence author. Mail address: No 111 Jiulong Road, Economic and Technology Development Zone, Hefei, Anhui, P. R. China 230,601, Tel: +86 551 63,861,441, Fax: +86 551 63,861,724. Email: 20023@ahu.edu.cn

This file includes:

Supplementary notes 1-5

Tables S1 to S4

Figures S1 to S6

Tables:

Table S1. Summary of Sequencing Data and BioProject Information.

Table S2. MAG Assembly Quality and Abundance in Samples.

Table S3. Primers for Target Genes.

Table S4. Landfill leachate physical and chemical indicators.

Figures:

Figure S1. Concentration of EPS in the biofilm, showing the dynamics of EPS concentration on days 15, 36, and 60 of formation.

Figure S2. Bacterial community composition at the phylum level, showing the top 10 phyla in different samples on 15, 36, and 60 days of formation.

Figure S3. Heatmap shows the top 20 pathogens in biofilms after Z-score normalization of species abundance data.

Figure S4. Dynamics of ARGs. (a) Composition of MLS ARGs in biofilms, with different colors representing different MLS ARGs. (b) Composition of Aminoglycoside ARGs in PVC and CS biofilms, with different colors representing different aminoglycoside ARGs.

Figure S5. Dynamics of ARGs(qPCR). (a) Dynamics of ermF ARG in PVC and CS biofilms. (b) Dynamics of *Campylobacter jejuni*, indicated by marker gene cadF in PVC and CS biofilm.

Figure S6. Procrustes analysis and bubble heatmap of ARGs in different MAGs. (a) Procrustes analysis of the relationship between Potential Pathogen and Microbiota. (b) Correspondence between metagenome-assembled genomes (MAGs) and ARGs. The

plot shows the relationship between MAGs and ARGs.The size of the dots indicates the abundance of ARGs within each MAG.

Supplementary material and method 1 Quantification of ARGs and MGEs by qPCR

DNA samples were prepared at a concentration of 2 ng/μL, and the PCR reaction mix contained 1× LightCycler 480 SYBR Green I Master, 500 nM of each primer, and the DNA template. Each sample was analyzed in triplicate, with ultrapure water used as a negative control. The reaction mix was dispensed into the SmartChip using the MSND system in a 64 (samples) × 80 (assays) format, with a final reaction volume of 100 nL per well. The PCR program included an initial denaturation step at 95°C for 10 minutes, followed by 40 cycles of 95°C for 30 seconds and 60°C for 30 seconds. A melting curve analysis was conducted at the end of the amplification cycles to confirm the amplification specificity. Ct values were automatically analyzed using the SmartChip qPCR software (version 2.7.0.1), with a detection threshold set at $C_t = 31$. Only samples with $C_t \leq 31$ that passed curve fitting analysis were considered positive for gene detection.

Supplementary notes 1 Biofilm chemical composition comparison

Initially, both P-15 and C-15 samples had similar mineral compositions, primarily CaO and $CaMg(Si_2O_6)$. Over time, the primary components of the biofilms changed, with notable differences observed between the mid (P-36 and C-36) and late (P-60 and C-60) stages. By Day 36, P-36 and C-36 demonstrated increased complexity, with additional compounds such as $Ca_3(Si_3O_9)$ on PVC and unique compounds like LiH on CS, suggesting distinct biofilm development dynamics due to substrate material adhesion. On Day 60, P-60 and C-60 exhibited the highest complexity, with new chemical compounds appearing on the biofilms ($CaCO_3(H_2O)$ on PVC and NH_4I on CS).

Gong et al. analyzed the chemical composition of biofilms in both aerobic and anaerobic systems in a leachate treatment plant, identifying O, Ca, and Mg as major components, along with K, Cl, and Bi, which is consistent with the biofilm chemical elements identified in this study [1]. By contrast, biofilms from a US landfill leachate contained Fe, calcium compounds, organic matter, carbonate, SiO_2, sulfide, and fine clays [2]. The differences between these biofilms also showed that variations in the physicochemical properties of the leachate also could cause the biofilm structure difference.

Supplementary notes 2 Biofilm microbiota composition

Similar to the biofilm bacterial phyla formed on MPs in leachate [3], Proteobacteria was the major group, occupying a significant ecological niche owing to its motility and metabolic versatility [4]. The proportion of Bacteroidetes increased with biofilm maturation, likely because of their ability to degrade organic matter, which facilitates the colonization of other bacteria [5]. Additionally, Actinobacteria were abundant on the biofilms owing to their resilience and ability to produce various bioactive compounds, including antibacterial, antifungal, and antiviral agents [6]. Firmicutes had a higher relative abundance in leachate (4.09%) than biofilms (0.49%-0.73%), possibly because the leachate environment provides abundant complex organics that favor their proliferation [7, 8].

Supplementary notes 3 Co-occurrence network analysis of biofilm key species

Among these, the Proteobacteria (CS: 75.11%, PVC: 69.74%), Actinobacteria (9.12%, 13.82%), and Bacteroidetes (8.25%, 9.24%) emerged as the most abundant taxa of keystone species, which are widely recognized as shared host bacteria for ARGs in biofilms [9, 10]. In addition, four species (*Nitrosomonas stercoris*, *Denitrificimonas caeni*, *Alcaligenaceae bacterium*, and *Parapusillimonas sp. SGNA-6*) were found to have a high number of connections (edges) in both co-occurrence networks, indicating that these species are highly interactive within the microbial community. All of them belong to the Proteobacteria, which was identified as one of the dominant groups in the biofilm networks. The abundance of connections suggests that these species may play important roles in the stability and functionality of the biofilms, acting as keystone species by facilitating various mutualistic or synergistic interactions with other microbial taxa.

Supplementary notes 4 Biofilm ARGs hosts information

Initially, significant differences were observed in the host species between CS and PVC biofilms. For example, *Stenotrophomonas maltophilia* and *Bacillota bacterium* were present on PVC but absent on CS biofilms at Day 15. Similarly, *Clostridia bacterium* was observed exclusively on PVC at this early stage. By Day 36, the host species began to converge. Species such as *Halopseudomonas xiamenensis* and *Acinetobacter haemolyticus* began appearing on both biofilms, indicating a trend toward uniformity in host-species composition. By Day 60, the convergence of host species on both biofilms was even more pronounced. These hosts included *Gammaproteobacteria bacterium*, *Acinetobacter baumannii*, *Klebsiella variicola*, *Pseudomonadota bacterium*, *Pseudomonas aeruginosa*, and *Proteus mirabilis.*

Supplementary notes 5 MAGs -based species host ARGs

The primary resistance mechanisms included antibiotic efflux, target protection, target replacement, and antibiotic inactivation, with efflux pumps being the most common mechanism. Genes such as macB, oleC, and tetA frequently appeared across multiple MAGs, indicating their widespread presence in the environment.

Species classification showed that these MAGs spanned various bacterial phyla, including Pseudomonadota (MAG31), Desulfobacterota (MAG339 and MAG146), Bacteroidota (MAG109 and MAG341), Bacillota (MAG295), Chloroflexota (MAG58), and Actinomycetota (MAG369). Moreover, the ARG profiles varied significantly among different MAGs. Specifically, MAG31 and MAG109 showed high abundances of tetracycline and macrolide resistance genes, whereas MAG339 and MAG146 contained numerous ARGs, particularly against fluoroquinolones and sulfonamides. MAG146 exhibited notable diversity and quantity of ARGs, including macB, oleC, tetA, vanR, and bcrA, demonstrating broad antibiotic resistance. High frequencies of macrolide resistance genes were detected in MAG58 and MAG369, revealing strong survival capabilities under macrolide selection pressure. The most common ARGs across different MAGs were macB, oleC, and tetA, suggesting their widespread occurrence in various environments and species. The ARG macB primarily confers resistance to macrolides, oleC is associated with macrolide secretion, and tetA is related to tetracycline efflux.

Table S1. Summary of Sequencing Data and BioProject Information.

Samples	group	Insert Size (bp)	Read length (bp)	Raw reads	Raw base (bp)	Clean reads	Clean base(bp)	Percent in raw reads (%)	Percent in raw bases (%)	BioProject ID
C_15_3	C15	500	150	90890740	1.37E+10	89392492	1.35E+10	98.35159	98.26316	PRJNA1108013
C_15_2	C15	500	150	1.09E+08	1.65E+10	1.07E+08	1.62E+10	98.49932	98.40386	PRJNA1108013
C_15_1	C15	500	150	91684952	1.38E+10	89795632	1.35E+10	97.93933	97.84166	PRJNA1108013
C_36_3	C36	500	150	1.01E+08	1.53E+10	99504904	1.5E+10	98.32551	98.23316	PRJNA1108013
C_36_2	C36	500	150	1.26E+08	1.91E+10	1.24E+08	1.88E+10	98.47273	98.38468	PRJNA1108013
C_36_1	C36	500	150	1.02E+08	1.55E+10	1.01E+08	1.52E+10	98.29486	98.20183	PRJNA1108013
C_60_1	C60	500	150	1.14E+08	1.72E+10	1.11E+08	1.68E+10	98.09882	97.99641	PRJNA1108013
C_60_3	C60	500	150	1.15E+08	1.74E+10	1.13E+08	1.71E+10	98.41711	98.32394	PRJNA1108013
C_60_2	C60	500	150	90241178	1.36E+10	88540102	1.34E+10	98.11497	98.00623	PRJNA1108013
L_1_2	L1	500	150	99955286	1.51E+10	97922812	1.48E+10	97.96662	97.87157	PRJNA1108013
L_1_3	L1	500	150	99243426	1.5E+10	97454196	1.47E+10	98.19713	98.10271	PRJNA1108013
L_1_1	L1	500	150	86939052	1.31E+10	85254908	1.29E+10	98.06285	97.96815	PRJNA1108013
P_15_2	P15	500	150	1.01E+08	1.53E+10	99724964	1.5E+10	98.30764	98.21669	PRJNA1108013
P_15_1	P15	500	150	1.32E+08	1.99E+10	1.3E+08	1.96E+10	98.39976	98.30643	PRJNA1108013
P_15_3	P15	500	150	1.18E+08	1.78E+10	1.16E+08	1.75E+10	98.29678	98.207	PRJNA1108013
P_36_1	P36	500	150	1.06E+08	1.6E+10	1.04E+08	1.58E+10	98.34946	98.25897	PRJNA1108013
P_36_2	P36	500	150	1.05E+08	1.59E+10	1.04E+08	1.56E+10	98.36591	98.27892	PRJNA1108013
P_36_3	P36	500	150	1.08E+08	1.63E+10	1.06E+08	1.6E+10	98.14777	98.05554	PRJNA1108013
P_60_3	P60	500	150	1.21E+08	1.83E+10	1.19E+08	1.8E+10	98.36577	98.26817	PRJNA1108013
P_60_1	P60	500	150	98780542	1.49E+10	96980796	1.46E+10	98.17804	98.06098	PRJNA1108013
P_60_2	P60	500	150	1.04E+08	1.57E+10	1.02E+08	1.54E+10	98.36398	98.24024	PRJNA1108013

Table S2. MAG Assembly Quality and Abundance in Samples.

MAG Id	Genome size	Scaffolds Num	Longest scaffold	N50 (scaffolds)	Mean scaffold length	Completeness	Contamination	Strain heterogeneity	Quality score	Quality
MAG146	2747882	56	196216	67052	49069.32	92.9	0.81	50	88.85	HQ
MAG109	2918690	116	88731	33688	25161.12	82.67	1.06	20	77.37	MQ
MAG339	2183132	76	172451	45157	28725.42	67.86	0	0	67.86	MQ
MAG31	1827417	22	459135	138420	83064.41	67.35	0.47	0	65	MQ
MAG341	1742014	26	340089	88763	67000.54	60.34	0	0	60.34	MQ
MAG295	1052742	186	28696	6711	5659.903	59.5	8.46	82.05	17.2	MQ
MAG58	1824008	212	60532	22318	8603.811	54.15	9.01	100	9.1	MQ
MAG369	1180413	319	12829	4458	3700.354	50.01	7.33	100	13.36	MQ

Table S3. Primers for Target Genes.

Gene type	Gene	Forward and Reverse
Sulfonamide	sul1	CAGCGCTATGCGCTCAAG
		ATCCCGCTGCGCTGAGT
	sul2	TCATCTGCCAAACTCGTCGTTA
		GTCAAAGAACGCCGCAATGT
Integron	intI-1	CGAACGAGTGGCGGAGGGTG
		TACCCGAGAGCTTGGCACCCA
MLS	ermF	CAGCTTTGGTTGAACATTTACGAA
		AAATTCCTAAAATCACAACCGACAA
Campylobacter jejuni	cadF	TGCTATTAAAGGTATTGATGTRGGTGA
		GCAGCATTTGAAAAATCYTCAT

Table S4. Landfill leachate physical and chemical indicators.

Indicator	Concentration
TN	2413.1 mg/L
TP	6.0 mg/L
NH_4^+-N	2366.0 mg/L
Nitrate Nitrogen	4.1 mg/L
Nitrite Nitrogen	0.6 mg/L
COD	1972.6 mg/L
BOD_5	55.1 mg/L
pH	8.0
ORP	-193.7 mV
Conductivity	27.2 mS/cm
Suspended Solids	156.5 mg/L
EPS	75.8 mg/L
DO	0.2 mg/L

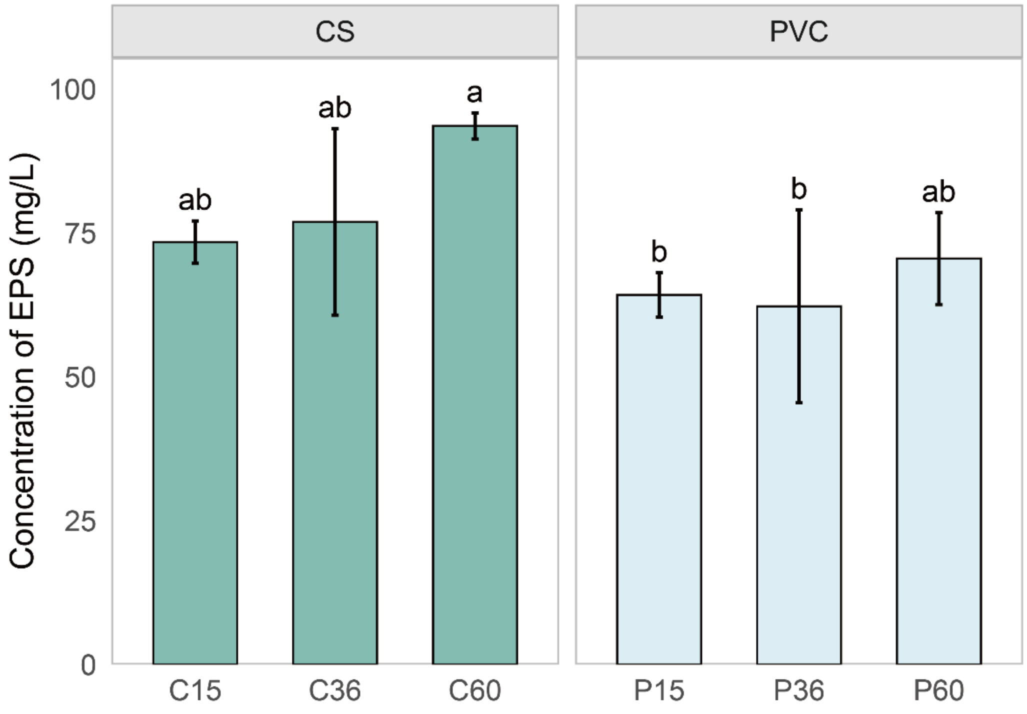

Figure S1. Concentration of EPS in the biofilm, showing the dynamics of EPS concentration on days 15, 36, and 60 of formation.

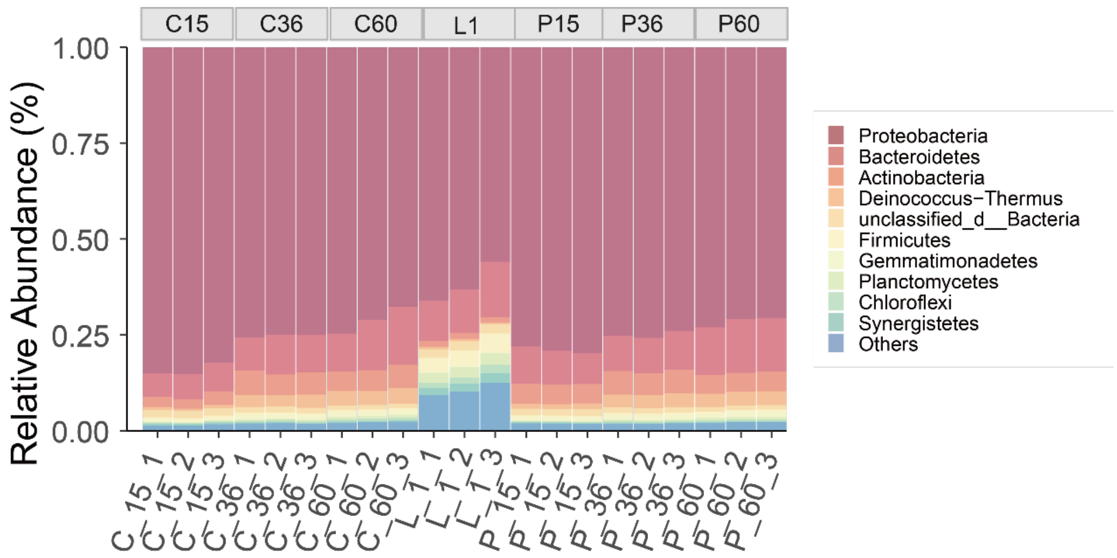

Figure S2. Bacterial community composition at the phylum level, showing the top 10 phyla in different samples on 15, 36, and 60 days of formation.

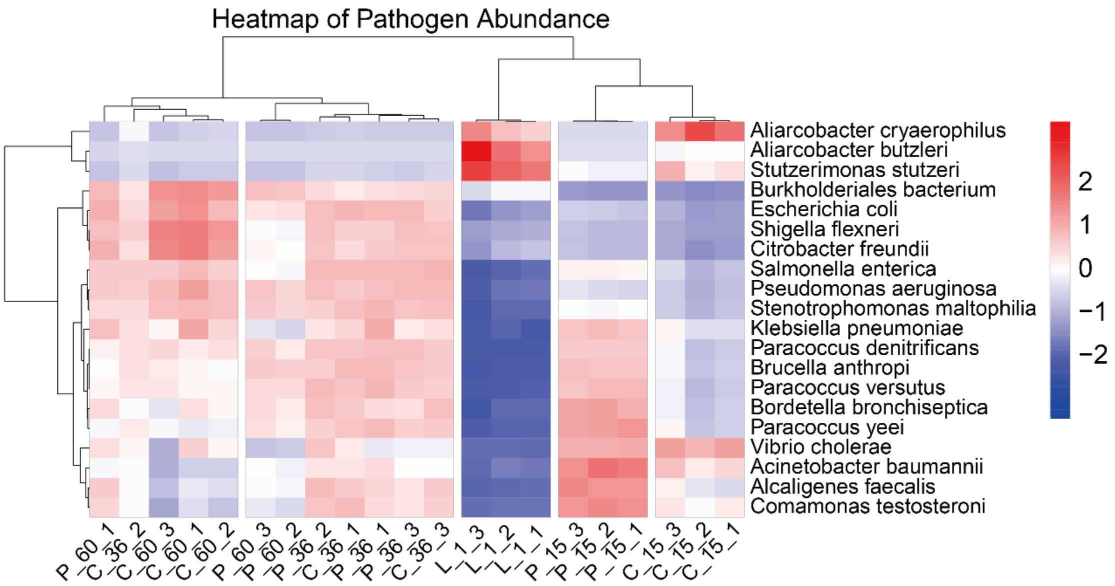

Figure S3. Heatmap shows the top 20 pathogens in biofilms after Z-score normalization of species abundance data.

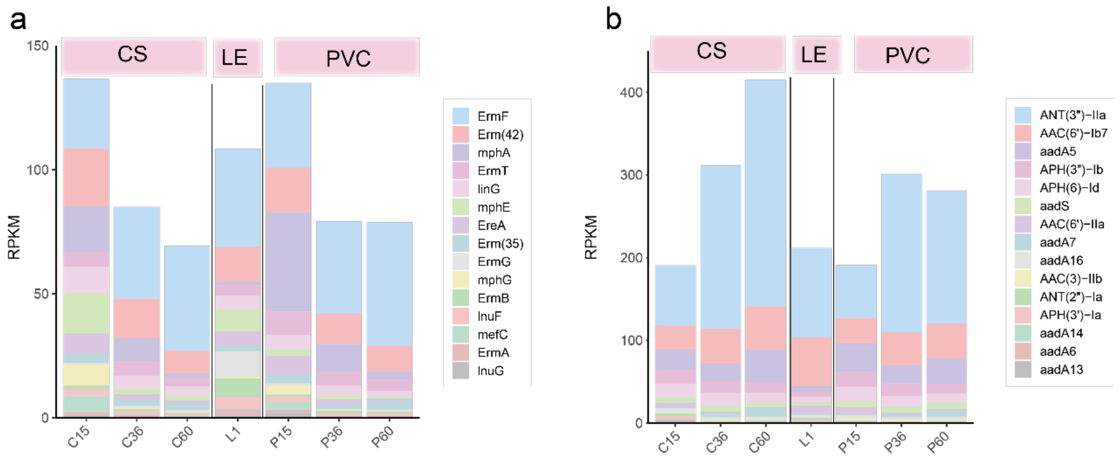

Figure S4. Dynamics of ARGs. (a) Composition of MLS ARGs in biofilms, with different colors representing different MLS ARGs. (b) Composition of Aminoglycoside ARGs in PVC and CS biofilms, with different colors representing different aminoglycoside ARGs. LE: leachate.

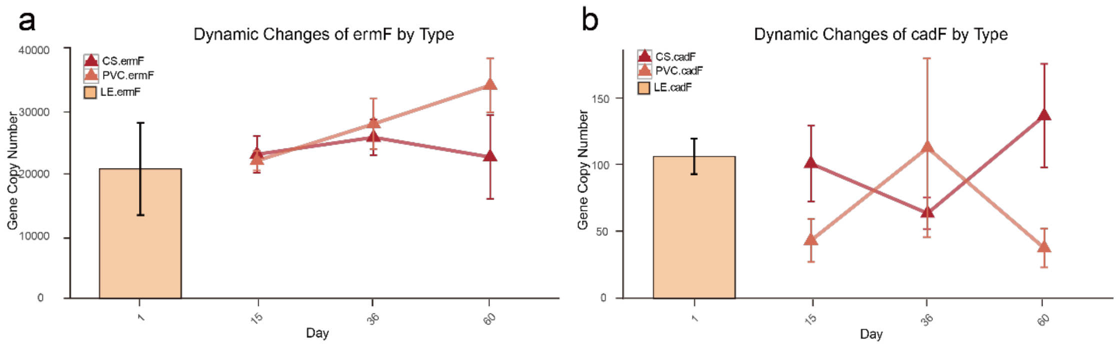

Figure S5. Dynamics of ARGs(qPCR). (a) Dynamics of ermF ARG in PVC and CS biofilms. (b) Dynamics of *Campylobacter jejuni*, indicated by marker gene cadF in PVC and CS biofilm.

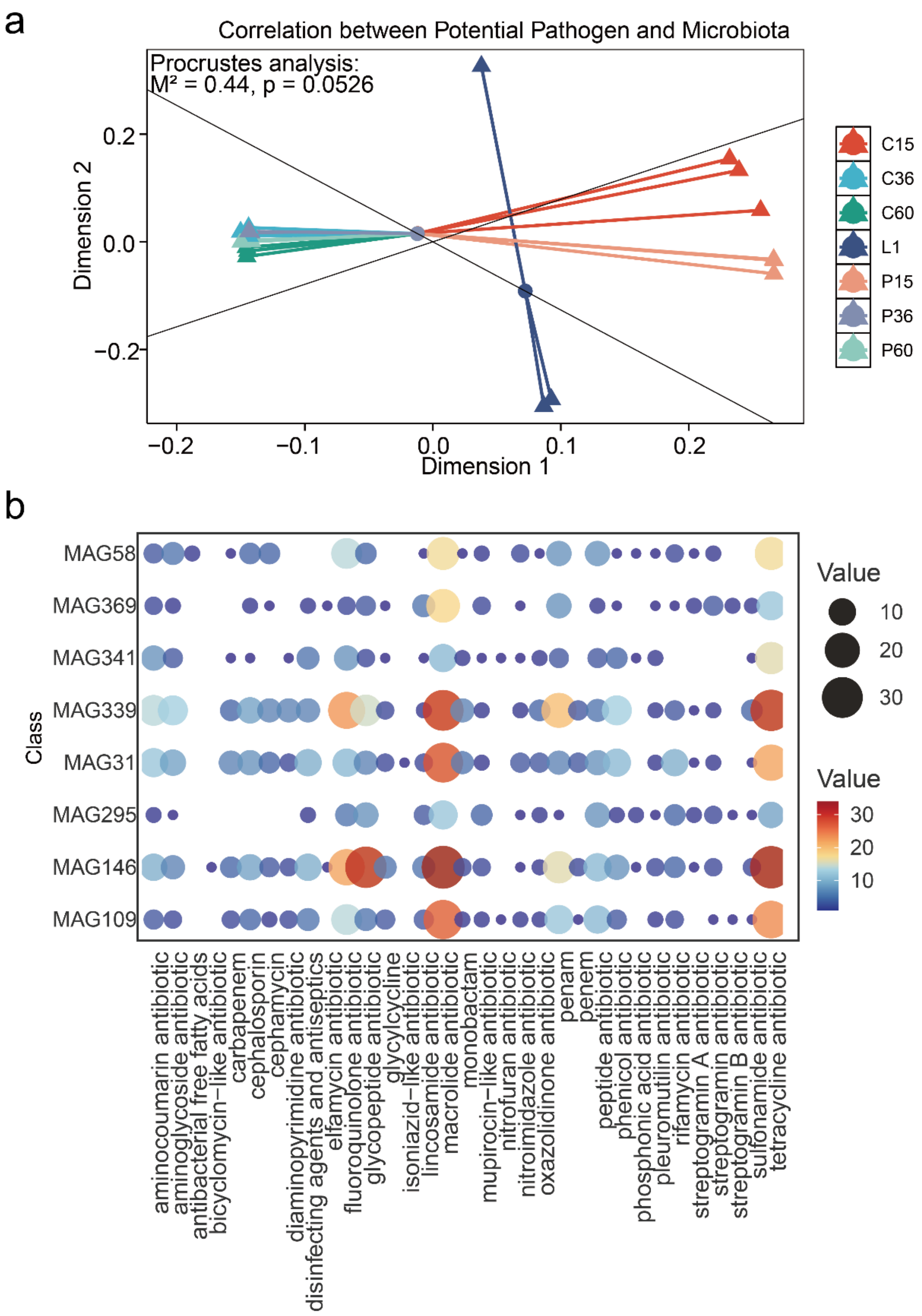

Figure S6. Procrustes analysis and bubble heatmap of ARGs in different MAGs. (a) Procrustes analysis of the relationship between Potential Pathogen and Microbiota. (b) Correspondence between metagenome-assembled genomes (MAGs) and ARGs. The plot shows the relationship between MAGs and ARGs.The size of the dots indicates the

abundance of ARGs within each MAG.

References

1. Gong Z, Yang S, Zhang R, Wang Y, Wu X, Song L. Physiochemical and biological characteristics of fouling on landfill leachate treatment systems surface. *J Environ Sci (China)*. 2024; 135:59-71.

2. Li Z, Xue Q, Liu L, Li J. Precipitates in landfill leachate mediated by dissolved organic matters. *J Hazard Mater*. 2015; 287:278-86.

3. Su Y, Zhang Z, Zhu J, Shi J, Wei H, Xie B, *et al.* Microplastics act as vectors for antibiotic resistance genes in landfill leachate: The enhanced roles of the long-term aging process. *Environ Pollut*. 2021; 270:116278.

4. Xiao S, Zhang Y, Wu Y, Li J, Dai W, Pang K, *et al.* Bacterial community succession and the enrichment of antibiotic resistance genes on microplastics in an oyster farm. *Mar Pollut Bull*. 2023; 194:115402.

5. Thomas F, Hehemann JH, Rebuffet E, Czjzek M, Michel G. Environmental and gut bacteroidetes: The food connection. *Front Microbiol*. 2011; 2:93.

6. Azman AS, Mawang CI, Khairat JE, AbuBakar S. Actinobacteria-a promising natural source of anti-biofilm agents. *Int Microbiol*. 2019; 22:403-09.

7. Dobrzyński J, Wróbel B, Górska EB. Taxonomy, ecology, and cellulolytic

properties of the genus bacillus and related genera. *Agriculture (Basel)*. 2023; 13:1979.

8. Jiang N, Huang L, Huang M, Cai T, Song J, Zheng S, *et al.* Electricity generation and pollutants removal of landfill leachate by osmotic microbial fuel cells with different forward osmosis membranes. *Sustain Environ Res*. 2021; 31:22.

9. Chen Y, Yan Z, Zhou Y, Zhang Y, Jiang R, Wang M, *et al.* Dynamic evolution of antibiotic resistance genes in plastisphere in the vertical profile of urban rivers. *Water Res*. 2024; 249:120946.

10. Wang J, Qin X, Guo J, Jia W, Wang Q, Zhang M, *et al.* Evidence of selective enrichment of bacterial assemblages and antibiotic resistant genes by microplastics in urban rivers. *Water Res*. 2020; 183:116113.
